# Triplanar Point Cloud Reconstruction of Head Skin Surface from Computed Tomography Images in Markerless Image-Guided Surgery

**DOI:** 10.3390/bioengineering12050498

**Published:** 2025-05-08

**Authors:** Jurica Cvetić, Bojan Šekoranja, Marko Švaco, Filip Šuligoj

**Affiliations:** Department of Robotics and Production System Automation, Faculty of Mechanical Engineering and Naval Architecture, University of Zagreb, 10002 Zagreb, Croatia; jurica.cvetic@fsb.unizg.hr (J.C.); bojan.sekoranja@fsb.unizg.hr (B.Š.); marko.svaco@fsb.unizg.hr (M.Š.)

**Keywords:** computed tomography, skin layer extraction, point cloud, markerless image-guided surgery

## Abstract

Accurate preoperative image processing in markerless image-guided surgeries is an important task. However, preoperative planning highly depends on the quality of medical imaging data. In this study, a novel algorithm for outer skin layer extraction from head computed tomography (CT) scans is presented and evaluated. Axial, sagittal, and coronal slices are processed separately to generate spatial data. Each slice is binarized using manually defined Hounsfield unit (HU) range thresholding to create binary images from which valid contours are extracted. The individual points of each contour are then projected into three-dimensional (3D) space using slice spacing and origin information, resulting in uniplanar point clouds. These point clouds are then fused through geometric addition into a single enriched triplanar point cloud. A two-step downsampling process is applied, first at the uniplanar level and then after merging, using a voxel size of 1 mm. Across two independent datasets with a total of 83 individuals, the merged cloud approach yielded an average of 11.61% more unique points compared to the axial cloud. The validity of the triplanar point cloud reconstruction was confirmed by a root mean square (RMS) registration error of 0.848 ± 0.035 mm relative to the ground truth models. These results establish the proposed algorithm as robust and accurate across different CT scanners and acquisition parameters, supporting its potential integration into patient registration for markerless image-guided surgeries.

## 1. Introduction

Robot-assisted image-guided surgeries (IGS) integrate imaging modalities like computed tomography (CT) and magnetic resonance imaging (MRI) with surgical procedures to enhance precision and minimize invasiveness. By reconstructing 3D volumes from 2D slices, IGS enables accurate preoperative planning and intraoperative tracking and is applied in fields such as cardiology, oncology, and neurosurgery [1]. IGS is categorized into marker-based and markerless approaches. While marker-based methods rely on fiducial markers for precise alignment [2], markerless approaches utilize anatomical landmarks and advanced imaging techniques to achieve alignment without the need for external markers, enhancing patient comfort and reducing recovery time. These methods are classified into three categories: anatomy-based, surface-based, and computer vision-based methods, each employing different techniques to improve registration accuracy [3]. However, accurate patient localization in markerless IGS remains challenging. A central issue is the reliable segmentation of the outer skin contour from 2D CT slices to create an accurate 3D representation, as optical sensors in the operating room capture the same anatomical data used for registration. Existing methods often rely on a single imaging plane [4], limiting spatial completeness and reducing registration accuracy. Moreover, variations in CT scanners, acquisition protocols, and patient anatomies can introduce inconsistencies in the reconstructed surface. These factors underscore the need for a robust algorithm that integrates information from axial, sagittal, and coronal slices to generate denser, more accurate point clouds and enhance registration in surgical applications.

This study addresses the challenge of generating a high-fidelity 3D skin surface from head CT scans by independently processing axial, sagittal, and coronal slices to capture comprehensive spatial information in order to obtain patient-specific point clouds from medical images. Binary images are obtained by applying thresholding within a defined Hounsfield unit (HU) soft tissue range; it was followed by the extraction of valid contours while eliminating data containing non-patient-related artifacts. These contours are constructed into uniplanar point clouds, which are merged into a single enriched triplanar point cloud. The algorithm’s performance is evaluated on two independent datasets. It demonstrates robustness across varying CT scanner models and acquisition parameters, which ensures accurate segmentation for precise image registration in image-guided applications.

This paper presents the following novel contributions:

- This work presents a novel outer skin layer extraction method from CT scans capable of handling external object interference. Each valid slice’s contour points are projected into 3D space using slice metadata to form uniplanar point clouds, which are fused via geometric addition into a triplanar point cloud to offer enhanced density and detail.
- To the best of our knowledge, no existing studies focus on extracting only the skin surface using DICOM metadata for registration purposes. We propose a novel approach that addresses this gap, resulting in a triplanar point cloud that structurally and visually resembles those captured intraoperatively by 3D cameras. This similarity enhances mutual compatibility and enables precise and robust markerless image-to-patient registration.

The paper is organized as follows: Section Related Work provides a literature review on skin and skull contour segmentation techniques and 3D visualization methods. Section 2 details the hardware and key software components used for valid contour segmentation and enriched point cloud generation. Section 3 presents the validation of the proposed algorithm on two datasets. Section 4 provides a discussion of the proposed method and its implications, while Section 5 presents the concluding remarks.

### Related Work

Methods for segmenting skin or skull contours from CT scans can be categorized into two groups: traditional image processing methods and machine/deep learning-based methods. Traditional methods rely on techniques such as thresholding, edge detection, and contour modeling. These approaches are straightforward and computationally efficient. In contrast, machine learning methods offer robustness in handling complex patterns, but in the case of deep learning, they require large labeled datasets for training and may fail in generalization. Although our primary focus is the underexplored area of skin layer extraction, we also examine studies on skull layer extraction, a more widely researched topic that addresses a related yet distinct problem.

For example, Ulinuha et al. extracted the skull contour using intensity-based thresholding and noise removal; it was followed by image scanning from four sides to identify the outer contour [5]. In a follow-up study, they applied the same method to segment skull and face contours, which were then reconstructed into point clouds [4]. The authors highlight the presence of medical equipment artifacts, such as CT scanner equipment in the skull and face point clouds. To address these challenges, we introduce a tissue-based bounding box derived from axial boundary points that refine sagittal and coronal point clouds, thereby excluding non-patient-related artifacts. Wu et al. [6] experimented with different thresholds in 2D and 3D processing, combined with morphological operations. Head contours were extracted via region growth with the level set method for active contour modeling. However, long reconstruction times and the limitation of a global threshold for region growth led to multiple threshold values per grouped slices [7]. MRI data grouped by skull shape and segmented using Otsu thresholding improved accuracy by refining the skull cluster through pixel-based segmentation. A similar skull removal method for computed tomography angiography (CTA) images is described in [8], where a user selects a small region within the skull boundaries to initialize the region growth algorithm, removing corresponding pixels. Chen et al. [9] segmented the skull as a preprocessing step for active contour models, using binary masks to isolate the skull region; it was followed by active contour models for reconstruction. Nevertheless, the sensitivity of the seed algorithm to initial placement is a key limitation, as it can cause the active contour to converge to the wrong region.

To overcome this, connected component analysis is utilized to group adjacent pixels of similar intensity into connected regions, thus providing a more reliable foundation for contour extraction. Roser et al. [10] presented an automatic CT preprocessing method for skin dose simulation and X-ray projections. Scans are labeled by HU values and segmented via connected components, where the largest cluster defines the head contour. Canny edge detection extracts the outer skin. Hokmabadi et al. [11] filtered neonatal CT images, removed the neck, and applied histogram thresholding and morphological operations for connected component analysis. Skull extraction involved Otsu thresholding, while scalp extraction relied on coupled level sets. The largest connected component principle is also used in [12], where multiple thresholds were applied to select the optimal one based on the fewest connected components. Fuzzy C-means (FCM) clustering offers an alternative method and focuses on intensity-based grouping instead of spatial connectivity, as shown in [13]. The FCM algorithm segments the bone structure based on grayscale intensity, selecting the cluster with the highest data point count for skull segmentation.

Three-dimensional reconstruction is commonly performed using the Marching Cubes algorithm, as shown in [14]. CT images undergo thresholding, Fast Marching, anisotropic diffusion filtering, and a secondary threshold to isolate the scalp. Reconstruction involved merging 2D slices into a 3D model using the Marching Cubes algorithm. A similar method is shown in [15], where MRI images are processed to generate a 3D point cloud using the Marching Cubes algorithm for outer skin surface extraction. Yoo et al. [16] used this method for automatic coarse registration. However, due to speed and robustness issues, Lechelek et al. [17] presented a hybrid method, combining two reconstruction algorithms to merge the correct regions.

Several toolkits, built on previous methods, are now also available to facilitate both segmentation and visualization. For example, Tiwary et al. [18] used the Visualization Toolkit (VTK) for volume and surface renderings, segmenting skin, bone, and soft tissues based on HU values. Meshes were extracted using *vtkMarchingCubes*, while surfaces were generated with *vtkContourFilter*. However, the authors noted challenges with structures having similar HU values. Eley et al. [19] explored different MRI techniques and performed craniofacial bone segmentation using automated algorithms with the Insight Segmentation and Registration Toolkit (ITK). Segmentation methods included image denoising, intensity normalization, head mask generation, and skin removal. Gsaxner et al. [20] segmented volumetric data into skin and anatomical structures, generating meshes using the Marching Cubes algorithm to create point clouds of the skin surface. Mazzocchetti et al. [21] used depth maps to process CT scans with MIMICS software, isolating the head’s bone structure and removing vertebrae. Eight snapshots were taken, and depth maps were converted into point clouds, which were merged and simplified.

Compared to traditional methods, machine learning provides more adaptive segmentations. A neural network-based approach in [22] involves CT skull segmentations via thresholding, manual filtering, and downsampling and a 2D U-Net model for MRI skull segmentations. 3D skull reconstructions were visualized in MIMICS and 3-matics, with high similarity to ground-truth CT skull renderings. Matzkin et al. [23] used global thresholding on CT scans to extract bone structures, performing virtual craniectomy to create a dataset for neural network training. A graph neural network method in [24] used dilation, edge detection, and curvature-based corner detection to generate 3D skull models from CT slices. Zhang et al. [25] used ITK-SNAP to segment CT images, which was followed by machine learning for classification and generating 3D skull models. Hu et al. [26] combined threshold segmentation, CNN-based closure operations, and region growth for skull extraction, thus ensuring accurate segmentation through morphological operations. While machine learning methods offer adaptability to diverse anatomies, they require large datasets, extensive computational resources, and time-intensive preprocessing.

Traditional methods for contour extraction typically rely on thresholding and morphological operations (4–7, 10–13). However, morphological operations risk altering the original slice information, potentially introducing inaccuracies. In contrast, our approach directly segments soft tissue based solely on HU values from the original image data without any additional preprocessing. Valid skin contours are extracted through direct contour detection and subsequently filtered to ensure anatomical accuracy. This method ensures that the generated point cloud is composed entirely of true data points, which is critical for achieving low RMS registration error and results in precise markerless image-guided surgery. Regarding 3D reconstruction, existing methods usually apply the Marching Cubes algorithm (14–17, 20) to obtain outer skull or skin surfaces. While effective, the Marching Cubes algorithm can introduce interpolation errors that compromise fidelity to the original image. Our method overcomes this limitation by extracting valid contour points from each slice and reconstructing them into point clouds using DICOM metadata, thereby preserving true anatomical boundaries and eliminating interpolation artifacts. Furthermore, to the best of our knowledge, no prior work specifically targets only the outer skin layer extraction for registration applications, unlike previous studies that focus predominantly on skull extraction or skin extraction for visualization purposes. Importantly, our point clouds resemble intraoperative 3D camera data and enable high mutual compatibility, ensuring precise registration essential for accurate surgical guidance. For visualization, we utilized VTK to generate binary images and employed the Point Cloud Library (PCL) for efficient point cloud rendering. Table 1 compiles the studies referenced in the related work section to provide a structured overview that supports the context of our study.

## 2. Materials and Methods

**Figure 1:**
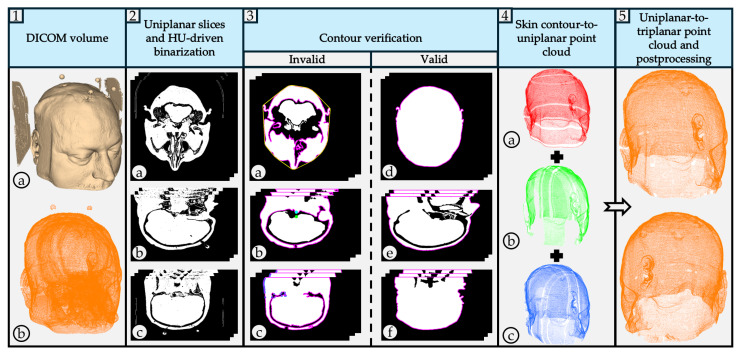
Outline of the proposed algorithm. An acquired DICOM volume corresponds to a single patient CT scan: 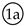 The CT scan model is processed in 3D Slicer using the CT soft tissue segmentation preset. Medical equipment artifacts and fiducial markers are visible. 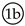 The 3D reconstructed point cloud, generated using the proposed algorithm, is presented without filters to emphasize external and intracranial noise. The acquired uniplanar slices (axial, sagittal, coronal) undergo HU-based thresholding to generate binary images: 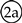 axial binary images; 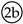 sagittal binary images; 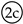 coronal binary images. The pseudocode is given in Algorithm 1. Valid contour extraction is performed on the binary images to delineate the skin layer. Several filters are applied to exclude images containing invalid contours: 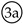 Contour solidity validation filter. Pink contour represents the largest found contour using OpenCV’s *findContours* function, while the yellow contour represents the contour’s convex hull area. The pseudocode is given in Algorithm 2. 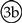 Four-direction contour validation filter. The pink contour represents the largest found contour. The blue circle represents the contour’s center of mass, while the green circle represents the pixel that satisfies both conditions presented in Algorithm 3. 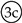 Pixel counting filter. The pink contour represents the largest found contour. The algorithm iterates over image rows in steps of 4, evaluating each column using an OpenCV method to identify contour pixels. A contour pixel is considered valid if it is at least 4 pixels away from the last detected one, incrementing a contour pixel counter. If this condition is met at least 4 times, a row pixel counter is also incremented. Images with the row pixel contour exceeding a threshold of 4 are excluded. Blue points indicate detected contour pixels. 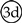 Valid axial slice contour. 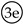 Valid sagittal slice contour. 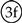 Valid coronal slice contour. Valid contours are used to generate 3D point cloud reconstructions from each view, generating three uniplanar point clouds: 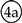 axial point cloud; 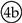 sagittal point cloud; 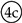 coronal point cloud. Generated uniplanar point clouds are merged into a triplanar point cloud to maximize structural detail and density. The triplanar point cloud is downsampled to eliminate redundant points and processed using two distinct outlier removal filters. 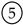 The same triplanar skin surface point cloud is presented from two perspectives, with medical equipment artifacts, fiducial markers, and intracranial noise eliminated.

Skin layer extraction is achieved through a four-step process: DICOM volume reslicing to obtain 2D slices, 2D slices thresholding to generate binary images, contour extraction to isolate valid structures, and point cloud generation for precise reconstruction. The outline of the proposed algorithm is illustrated in Figure 1.

CT images are used for creating binary images by employing the Visualization Toolkit (VTK 9.2.2.) and further processed using the Open Source Computer Vision Library (OpenCV 4.8.1.). The 3D reconstruction is performed using the Point Cloud Library (PCL 1.13.1.). For evaluation, ground truth (GT) models are generated using 3D Slicer (v5.6.2), while CloudCompare (v2.11.1) is utilized for point cloud-to-model registration, as well as further point cloud analysis. The proposed algorithm is developed in C++ using the Visual Studio Code editor (v1.98.2) on an HP Z4 G4 workstation (Intel Core i9-10920X, 3.50 GHz, NVIDIA Quadro RTX 5000, 64 GB RAM) running Ubuntu 22.04.5 LTS.

### 2.1. Data Preprocessing

All data are stored in DICOM format. Using VTK, DICOM data are loaded into a volumetric representation. To extract 2D slices, a systematic reslicing approach is employed. The slicing orientation is defined using a transformation matrix configured to align the slicing plane with the desired axis of the volume, depending on the selected view (axial, sagittal, or coronal). Based on the position of the initial slice and the slice spacing, slices are extracted throughout the volume. To apply image processing techniques, threshold-based segmentation is used to generate binary images of skin tissue from the extracted slices based on a defined HU range. CT values for the same body tissues can vary between different CT scanners and with varying energy levels on the same scanner, as noted in [27]. For this study, a range of −200 to +220 HU is used to isolate skin tissue. The range is determined heuristically by considering values suggested in [27,28], as well as through experimental visualization of soft tissues across various HU ranges in 3D Slicer. Each pixel intensity is retrieved and mapped to a binary value based on the defined threshold range, creating an image that highlights structures within this range. The pseudocode is given in Algorithm 1.
**Algorithm 1:** CT slice to binary mask conversion
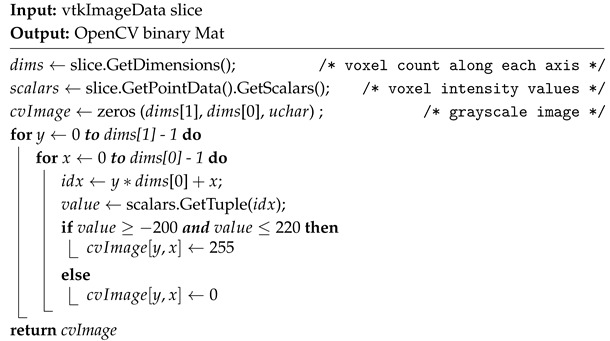


### 2.2. Image Processing

Previously obtained binary images are further refined to generate valid point clouds representing only the outer skin of each patient’s head, eliminating both external and internal noise. External noise is caused by medical equipment and varying medical wound care, which have HU values within the threshold range. Internal noise arises from anatomical differences, leading to disrupted contours. To mitigate these issues, multiple filters are applied. Unlike most studies that rely on morphological operations, this study avoids such preprocessing steps and directly extracts contours from raw data, thereby preserving original image integrity. The initial step involved detecting all contours within each image using OpenCV’s *findContours* function. Contours in specific image regions, identified as medical equipment artifacts, are removed based on their computed center of mass positions, with the X and Y center of mass coordinates compared to heuristically determined threshold values. If the coordinates fall outside the valid range, the image is excluded. Next, the remaining contours are sorted by area, and the largest contour is selected, corresponding to the outer skin boundary. To address intracranial noise caused by patient-specific anatomical variations, a pixel-connection method is employed. A line is drawn between the first and last appearing white pixels in the first image row to maintain region connectivity, preventing intracranial noise from interfering with contour extraction that may incorrectly include internal head structures. The newly found contours are again sorted by area, and the largest contour is retained. To preserve the integrity of the original data, the first row containing the connected pixels, located at the lower boundary of the head, is removed to prevent unintended alterations.

An established solidity filter is then applied to exclude contours that did not meet a predefined solidity threshold. First, the contour area and its corresponding convex hull area are computed. Solidity is then determined as the ratio of the contour area to its convex hull area, ranging from 0 to 1. Lower solidity values indicate greater contour irregularity. In this study, a threshold of 0.88 was applied, as it was heuristically determined by analyzing a wide range of contours from multiple patient slices across both datasets. Through this empirical observation, it was found that contours with solidity values above 0.88 corresponded to valid skin contours. Solidity was calculated for contours with areas exceeding 40,000 pixels, as contours within this range are expected to exhibit round and regular shapes. Images where contours show poor solidity are excluded, as this indicates the presence of intracranial noise due to complex patient anatomies. The pseudocode is given in Algorithm 2.
**Algorithm 2:** Contour Solidity Validation
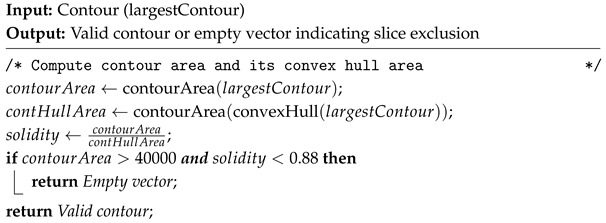


Next, the area difference between consecutive contours is calculated. If it falls within a predefined threshold, an additional filter removes slices where invalid contours fail to enclose the correct region. This filter is important in cases where the largest contour is misidentified due to discontinuities that exclude a significant portion of an area. The contour’s center of mass is used as a reference for scanning in four directions, which verifies the presence of white pixels outside the identified contour. If a white pixel appears outside the largest contour, the slice is discarded, as three imaging planes provide enough spatial data to justify this strict exclusion. The pseudocode is given in Algorithm 3.
**Algorithm 3:** Four-Direction Contour Validation
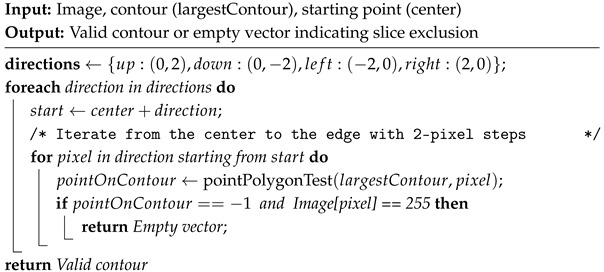


To further address smaller intracranial noise that may bypass the solidity filter, three complementary pixel counting filters are implemented. These focus on artifacts in anatomically complex regions, such as the ear and nose, where structural intricacies introduce noise. The filters iterate over rows and employ an OpenCV method to detect contour pixels at specific column intervals. If the number of detected contour pixels per row exceeds a predefined threshold, a counter is incremented. When the counter surpasses a set limit, the slice is excluded, as shown in Figure 1, 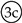. The final filter is applied based on heuristically determined locations of medical equipment in the lower region of the image.

Each pixel intensity within the region is checked, and if a white pixel is found, the slice is excluded. Region sizes are determined based on expected dimensions and positions of the head in binary images, which corresponded to specific contour areas.

### 2.3. Spatial Reconstruction

Contour points from valid slices are converted into a VTK PolyData structure to enable spatial reconstruction using DICOM metadata. Each point is mapped from image to world coordinates based on the volume’s spacing, origin, slice origin, and dimensions (number of slices) along each axis. The slice number serves as the third dimension, varying according to the slicing orientation. For sagittal and coronal views, the z-axis coordinates are adjusted to match the orientation of the axial point cloud. This allows all point clouds to share the same orientation, enabling their geometric addition. The pseudocode is presented in Algorithm 4.

Since the axial view produced point clouds with the least artifacts, it is used to establish acceptable minimum and maximum values for the X, Y, and Z coordinates in the 3D coordinate system, as scanner design and patient positioning minimize external obstructions in this standard imaging orientation. Points from the sagittal and coronal views are then subjected to defined thresholds to exclude external artifacts. These transformed points are subsequently converted into point cloud data using PCL. To remove redundant points, each point cloud is downsampled to a voxel size of 1 millimeter. The three resulting uniplanar point clouds are combined into a single enriched triplanar point cloud. The triplanar point cloud is then downsampled to the same voxel sizes, allowing for better determination and visualization of additional points obtained from sagittal and coronal point clouds. For positional accuracy analysis, the same process is repeated with a voxel size value of 0.38 mm, corresponding to the dataset’s minimum pixel spacing obtained from DICOM metadata. Finally, the triplanar point cloud is subjected to two different outlier removal filters, one based on local point density and the other on deviations from the mean, to eliminate any remaining external noise.
**Algorithm 4:** 2D Slice Contour Point to 3D Space
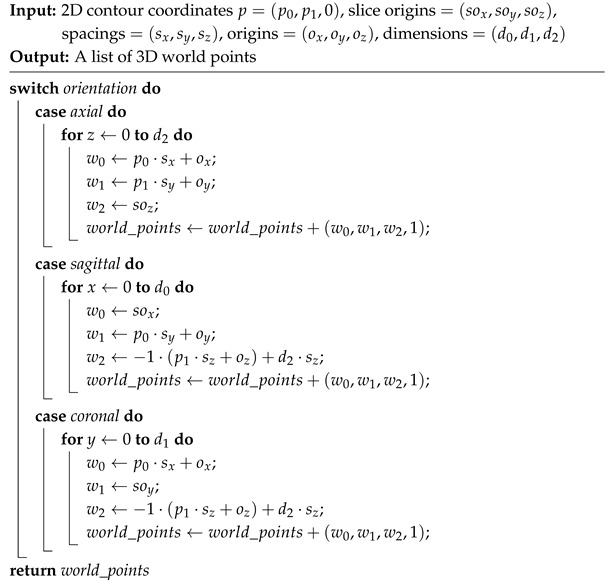


## 3. Results

### 3.1. Datasets

This study is evaluated on two different head CT datasets. The first utilized dataset was collected as a part of the research [29] on the RONNA system conducted at the Dubrava University Hospital, with prior approval from the hospital’s ethical committee and informed consent obtained from all patients involved. The dataset consists of 19 individuals (5 female, 14 male) with a median age of 55. The slice thickness varies between 0.6 and 0.75 mm. The second utilized and publicly available dataset is CQ500 [30]. The dataset consists of 491 anonymous individuals (178 female, 313 male) with an average age of 48.08 years. For purposes of the study, 64 random individuals were employed for the second dataset. The slice thickness is 0.625 mm. Both datasets were acquired using convolution kernels optimized for soft tissue imaging, with the image orientation set to the standard axial plane. The datasets were acquired using various CT scanner models, including the Siemens Somatom Emotion (Siemens Healthineers, Erlangen, Germany) at the University Hospital Dubrava, and, for the CQ500 dataset, the GE BrightSpeed, GE Discovery CT750 HD, GE LightSpeed, and GE Optima CT660 scanners (GE Healthcare, Chicago, IL, USA), as well as the Philips MX 16-slice and Philips Access-32 CT scanners (Philips Healthcare, Best, The Netherlands).

### 3.2. Identifying Unique Points

**Figure 2:**
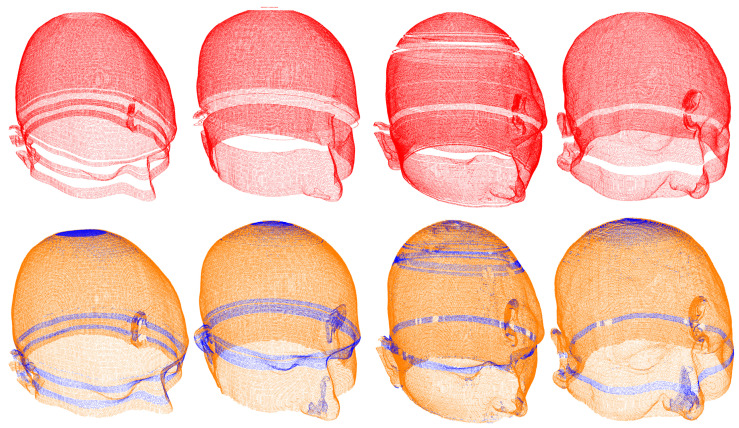
Each column represents an individual, with the first two columns representing the CQ500 dataset and the last two Dubrava University Hospital dataset. The top row represents the outer skin contour reconstruction using axial slicing, while the bottom row displays the triplanar reconstruction from three orthogonal views: axial, sagittal, and coronal. The blue points represent the unique difference points identified from sagittal and coronal views.

The acquired datasets served as inputs for the proposed algorithm. Numerical and visual information is obtained from both uniplanar and triplanar point clouds. A comparison of the triplanar point cloud is carried out with the axial cloud, as both datasets share the same axial imaging orientation. The mean number of additional points in the triplanar point cloud is 36 385, which corresponds to an average increase of 40.57%. The minimum and maximum increases are 16 368 (17.61%) and 74 039 (88.71%), respectively. To quantify the advantage of using three slicing views, K-d trees are utilized to compute the numerical difference in unique points between the axial and triplanar point clouds. A radius search method with a tolerance of 1 mm is implemented to identify the nearest neighbors of the triplanar cloud points in the axial cloud. On average, 15 089 points in the triplanar point cloud (11.61% of its total points) are unique, meaning they are absent in the axial cloud. The number of unique points varies between 3362 (2.51%) and 45 991 (28.24%). These results highlight the benefit of combining three slicing views to enrich uniplanar point clouds, as illustrated in Figure 2.

### 3.3. Positional Accuracy Analysis

**Figure 3:**
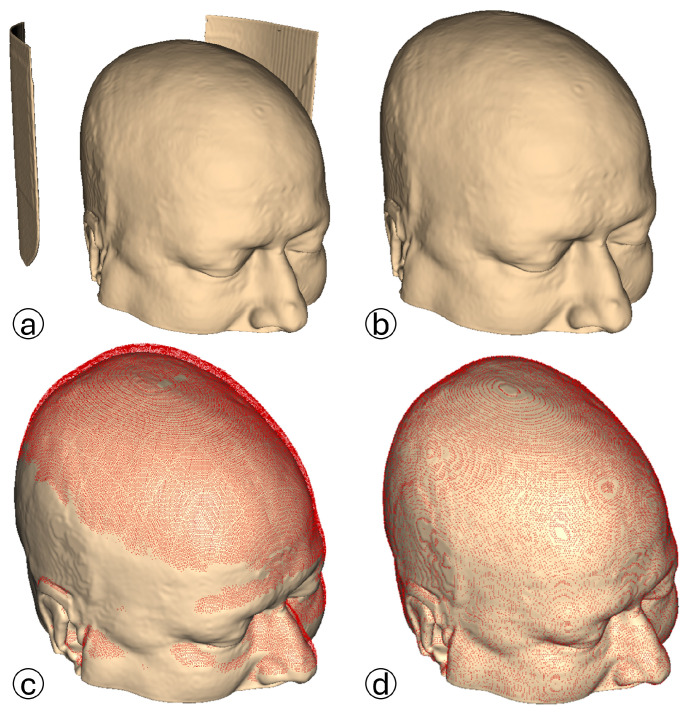
Proposed algorithm evaluation process: ⓐ Soft tissue segmentation based on HU values using 3D Slicer; ⓑ Medical equipment artifacts removal; ⓒ Manual alignment as a preprocessing step before the fine registration algorithm; ⓓ Fine registration. The red point cloud represents the reconstructed triplanar point cloud, while the skin color model represents the GT.

To evaluate the positional accuracy of valid contour points extracted by the proposed algorithm, we performed fine registration between the GT models and the reconstructed triplanar point clouds, as shown in Figure 3. GT for each scan is generated using 3D Slicer by applying the CT soft tissue segmentation preset (HU range available on the official 3D Slicer GitHub repository, https://github.com/Slicer/Slicer (accessed on 5 May 2025)), resulting in models that include both internal structures and the outer skin layer. The models are refined using the *Islands* function to eliminate noisy data, such as medical equipment artifacts. Reconstructed point clouds are imported into CloudCompare alongside their corresponding GT models. Downsampling is avoided to preserve the integrity of the GT data. Initial manual alignment is performed to facilitate fine registration via the Iterative Closest Point (ICP) algorithm.

ICP parameters are standardized across all datasets: RMS difference of 1 ×$10^{-4}$ mm, final overlap of 90%, and random sampling limited to the number of points in the reconstructed point cloud. The parameters are chosen to reflect the similarity between the GT model and the reconstructed point cloud, ensuring the use of all valid points for fine registration. Farthest point removal is enabled to ensure the registration of relevant points. Resultant RMS registration errors are saved and later used in the evaluation process.

**Figure 4:**
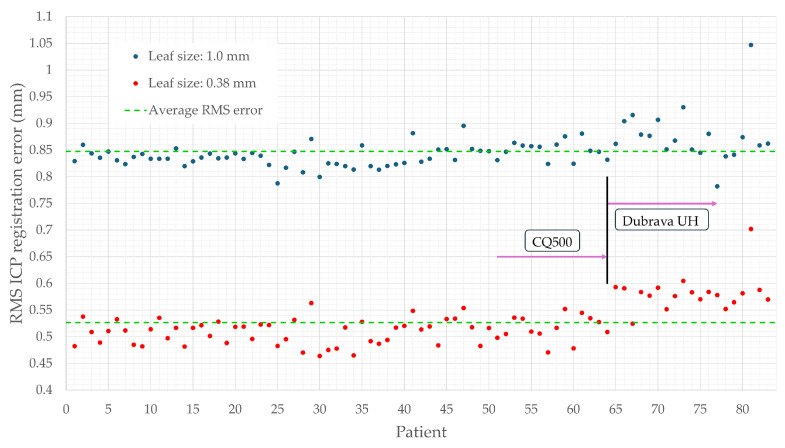
Root mean square (RMS) registration errors of reconstructed triplanar point clouds and GT models. Blue and red points indicate errors for point clouds downsampled to a voxel size of 1 mm and 0.38 mm, respectively. Each triplanar cloud was obtained by downsampling uniplanar clouds, merging them, and applying a second downsampling step. The green line represents the averaged RMS errors for both cases.

Fine registration is performed on two downsampled point cloud datasets. A voxel size of 1 mm is selected to retain essential details while eliminating redundancy. The 0.38 mm voxel size, derived from DICOM metadata, represents the minimum pixel spacing, below which further downsampling provides no significant benefit. Point clouds downsampled to a 1 mm voxel size have a mean RMS registration error of 0.848 ± 0.035 mm, with values between 0.782 mm and 1.047 mm. Point clouds downsampled to a 0.38 mm voxel size show a mean error of 0.526 ± 0.040 mm, ranging from 0.463 mm to 0.702 mm, as shown in Figure 4. The proposed algorithm demonstrated robustness to variations in CT scanners and their parameters. The highest RMS error is observed in a patient from the Dubrava University Hospital dataset with fiducial markers fixed to the head. Although a few patients had attached fiducial markers, the algorithm effectively filtered out noise, ensuring accurate reconstructions.

### 3.4. Assessing Triplanar Point Cloud Density

**Figure 5:**
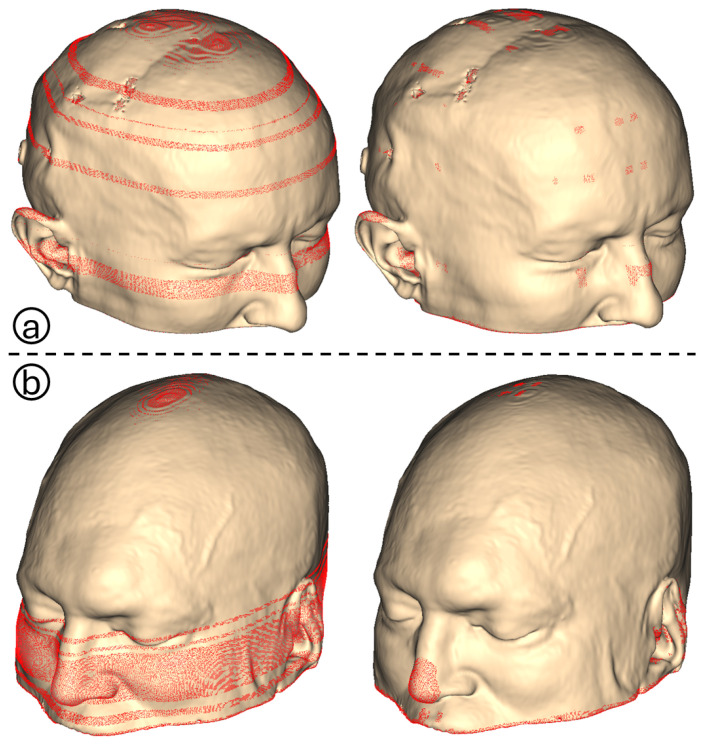
Cloud/Cloud distance tool results: ⓐ Patient 1; ⓑ Patient 2. The red points in the left images represent the areas of missing points in the axial point clouds, while the right images display the areas of missing points in reconstructed triplanar point clouds. The skin color models represent the GT.

The completeness of the reconstructed point cloud is visually assessed using the Cloud-to-Cloud distance tool in CloudCompare. Axial and triplanar point cloud vertices undergo separate registration with the GT model before being used as inputs for scalar field computation. The scalar fields are configured to highlight points of the GT model that are at least one millimeter away from point cloud vertices, first in comparison with the axial point cloud and then with the triplanar point cloud, as illustrated in Figure 5. Visual inspection of the axial and triplanar scalar fields revealed a higher point density in the triplanar point clouds. The majority of missing areas in the triplanar point clouds are located near the ears, nasal tips, and cranial vertices. These regions are either excluded due to their complex anatomy, which the algorithm filtered out, or because noise present in the uniplanar point clouds in these areas was removed by discarding the entire slice. During image processing, the bottom row of each slice is discarded, and therefore, the points in the bottom red region are not considered in the analysis.

### 3.5. Execution Time

The algorithm achieved an average execution time of 28.55 s, with minimum and maximum execution times of 23.61 and 36.26 s, respectively. This variation is primarily influenced by differences in the number of slices across the CT scans.

## 4. Discussion

Although the work presented shows promising results, a limitation of the proposed method is the lack of dynamic adaptability across different CT scanners and patient anatomies. While the algorithm successfully processes data from multiple patient datasets, the filter parameters currently remain static, which may lead to suboptimal segmentation depending on convolution kernels, scanner models, imaging orientation, or anatomical variability. This constraint can result in a loss of segmentation detail that could otherwise enhance the quality of the reconstructed point cloud. To address this, future work will explore automatic parameter adjustment using machine learning models that leverage CT metadata, such as convolution kernel type and scanner-specific information. A learning-based optimization of filtering parameters could improve segmentation fidelity, resulting in denser and more anatomically accurate point clouds.

Regarding the methodology, we acknowledge that the pipeline primarily employs classical image processing techniques, including thresholding and contour extraction. Although deep learning approaches such as U-Net for image segmentation or PointNet for point cloud processing are increasingly popular, they typically require extensive annotated datasets that are difficult to obtain in medical imaging. Furthermore, manual labeling of CT skin surfaces is time-consuming and prone to interobserver variability. Importantly, erroneous segmentation by neural networks could introduce artifacts into the reconstructed point cloud, potentially increasing the RMS registration error and reducing surgical precision, which is critical in neurosurgical interventions. Therefore, we opted for a classical approach that maximizes transparency, minimizes computational resources, and preserves fidelity to the original image data by avoiding morphological operations that could distort anatomical structures.

In terms of real-world application, validation is planned using an adult head phantom compatible with CT imaging to simulate a preoperative setting. A CT scan of the phantom will be processed using our pipeline to extract the outer skin surface point cloud. Separately, a 3D camera will capture an intraoperative point cloud from the phantom surface. Registration between the two point clouds will then be performed, and the RMS registration error will be quantified. Additionally, fiducial markers embedded in the phantom can be used to independently measure registration accuracy. Achieving submillimeter RMS error would indicate sufficient precision for clinical translation.

Upon successful phantom validation, the method could be implemented in neurosurgical workflows to provide accurate preoperative surface models for registration with intraoperative data. This would facilitate markerless localization, enabling precise robotic navigation and operative planning without reliance on fiducial markers. Such an advancement would reduce surgical preparation time, eliminate marker placement-related patient morbidity, and improve overall workflow efficiency.

## 5. Conclusions

In this study, we introduce a novel algorithm for reconstructing the outer skin layer point cloud from head CT scans. The algorithm effectively removes intracranial noise and medical equipment artifacts by leveraging several image processing techniques, thus producing accurate outer skin layer point clouds. A K-d tree-based method identified unique difference points between the reconstructed triplanar and axial point clouds, revealing that, on average, 11.61% of the triplanar cloud’s points are unique and absent from the axial cloud. The reconstructed triplanar point cloud demonstrated an average RMS registration error of 0.848 ± 0.035 mm compared to the ground truth model, proving the algorithm’s accuracy and reliability in the reconstruction process. This paper presents a novel algorithm for outer skin layer extraction in the presence of external objects from CT scans, which enhances detail and number of points compared to uniplanar point clouds. The algorithm was tested on two datasets, demonstrating robustness across different CT scanners and acquisition parameters. Evaluation results suggest its potential for integration into markerless image-guided surgery systems, as it generates point clouds comparable to intraoperative data from 3D cameras or lasers. This integration could improve registration accuracy and preoperative planning efficiency.

## Figures and Tables

**Table 1:** Comparison of Related Works on Skull and Skin Segmentation and Reconstruction.

Study	Modality	Segmentation Method	Reconstruction/Output	ML/AI Used	Skin Extraction Focus
Ulinuha et al. [4,5]	CT	Thresholding + Scanning	Face + skull reconstruction	No	Yes, No
Wu et al. [6]	CT	Morphology + Region Growing + Level Set	Skull reconstruction	No	No
Tuan et al. [7]	MRI	Otsu Thresholding + Pixel Clustering	Skull segmentation	No	No
Zhou et al. [8]	CTA	Manual Seed + Region Growing	Skull pixel removal	No	No
Chen et al. [9]	CT	Binary Mask + Active Contour	Skull reconstruction	No	No
Roser et al. [10]	CT	HU Filtering + Canny Edge + Connected Components	Skin layer segmentation	No	Yes
Hokmabadi et al. [11]	MRI	Histogram Thresholding + Morphology + Coupled Level Sets	Skull + scalp segmentation	No	Yes
Dangi et al. [12]	CT	Multi-threshold + Connected Components	Skull segmentation	No	No
Gupta et al. [13]	CT + MRI	Fuzzy C-means Clustering	Bone structure segmentation	No	No
Dai et al. [14]	CT	Fast Marching + Thresholding + Filtering	Scalp reconstruction	No	Yes
Deepika et al. [15]	MRI	Thresholding + Marching Cubes	Skin reconstruction	No	Yes
Yoo et al. [16]	CT	Marching Cubes	Skin reconstruction	No	Yes
Lechelek et al. [17]	MRI	FE + MPU	3D brain reconstruction	No	No
Tiwary et al. [18]	CT	HU-based Segmentation (VTK)	Soft tissue + skull rendering	No	Yes
Eley et al. [19]	MRI	Automated Segmentation (ZTE)	3D craniofacial reconstruction	Yes	Yes
Gsaxner et al. [20]	CT	Studierfenster + Marching Cubes	Skin reconstruction	No	Yes
Mazzocchetti et al. [21]	CT	Depth Maps + MIMICS	3D merged point clouds	Yes	Yes
Vu et al. [22]	CT + MRI	Thresholding + U-Net	Skull reconstruction (MRI + CT)	Yes	No
Matzkin et al. [23]	CT	Thresholding + U-Net	Skull reconstruction	Yes	No
Sarmah et al. [24]	CT	Edge Detection + Curvature + GNN	3D skull models	Yes	No
Zhang et al. [25]	CT	Threshold + ML Classification	Skull reconstruction	Yes	No
Hu et al. [26]	CT	Threshold + CNN Closure + Morphology	Skull segmentation	Yes	No

## Data Availability

The code and data used in this study are available on GitHub at https://github.com/CRTA-Lab/ct2pcd (accessed on 5 May 2025).

## Abbreviations

The following abbreviations are used in this manuscript:

CT	Computed Tomography
RMS	Root Mean Square
IGS	Image-Guided Surgery
MRI	Magnetic Resonance Imaging
3D	Three-Dimensional
2D	Two-Dimensional
HU	Hounsfield Unit
CTA	Computed Tomography Angiography
FCM	Fuzzy C-Means
DICOM	Digital Imaging and Communications in Medicine
VTK	Visualization Toolkit
ITK	Insight Segmentation and Registration Toolkit
MIMICS	Materialise Interactive Medical Image Control System
PCL	Point Cloud Library
CNN	Convolutional Neural Network
OpenCV	Open Source Computer Vision Library
GT	Ground Truth
ICP	Iterative Closest Point
